# Performance in Neurocognitive Tasks in Obese Patients. Does Somatic Comorbidity Matter?

**DOI:** 10.3389/fpsyt.2013.00084

**Published:** 2013-08-13

**Authors:** Wibke Kiunke, Christina Brandl, Ekaterini Georgiadou, Kerstin Gruner-Labitzke, Thomas Horbach, Hinrich Köhler, Martina de Zwaan, Astrid Müller

**Affiliations:** ^1^Schoen Clinic Bad Bramstedt, Bad Bramstedt, Germany; ^2^Department of Psychosomatic Medicine and Psychotherapy, Friedrich-Alexander University of Erlangen-Nuremberg, Erlangen, Germany; ^3^Department of Psychosomatic Medicine and Psychotherapy, Hannover Medical School, Hannover, Germany; ^4^Department of Surgery, Herzogin Elisabeth Hospital, Braunschweig, Germany; ^5^Department of Surgery, Municipal Hospital Schwabach, Schwabach, Germany

**Keywords:** obesity, neurocognitive functioning, type 2 diabetes, hypertension, sleep apnea

## Abstract

The aim of the present study was to examine if obese individuals with obesity-related somatic comorbidity (i.e., hypertension, diabetes, sleep apnea, dyslipidemia, pain disorder) perform worse in neurocognitive tasks compared to obese individuals without any somatic disorder. Neurocognitive functioning was measured by a computerized test battery that consisted of the following tasks: Corsi Block Tapping Test, Auditory Word Learning Task, Trail Making Test-Part B, Stroop Test, Labyrinth Test, and a four-disk version of the Tower of Hanoi. The total sample consisted of 146 patients, the majority (*N* = 113) suffered from obesity grade 3, 26 individuals had obesity grade 2, and only 7 individuals obesity grade 1. Ninety-eight participants (67.1%) reported at least one somatic disorder (Soma^+^-group). Hypertension was present in 75 individuals (51.4%), type 2 diabetes in 34 participants (23.3%), 38 individuals had sleep apnea (26.0%), 16 suffered from dyslipidemia (11.0%), and 14 individuals reported having a chronic pain disorder (9.6%). Participants without a coexisting somatic disorder were younger [*M*_Soma−_ = 33.7, SD = 9.8 vs. *M*_Soma+_ = 42.7, SD = 11.0, *F*(1, 144) = 23.01, *p* < 0.001] and more often female [89.6 and 62.2%, χ^2^(1) = 11.751, *p* = 0.001] but did not differ with respect to education, regular binge eating, or depressive symptoms from those in the Soma^+^-group. The Soma^−^-group performed better on cognitive tasks related to memory and mental flexibility. However, the group differences disappeared completely after controlling for age. The findings indicate that in some obese patients increasing age may not only be accompanied by an increase of obesity severity and by more obesity-related somatic disorders but also by poorer cognitive functioning.

## Introduction

Research indicates an association between obesity and neurocognitive dysfunction (1–4). The causal pathways for this relationship, however, remain poorly understood. One potential explanation concerns the association between obesity and comorbid medical conditions that in turn may lead to cognitive impairment. It remains uncertain whether the proposed cognitive decline can be attributed to effects of a specific obesity-related somatic disorder or rather to additive effects of multiple conditions. Cognitive dysfunction seen in patients with obesity might be caused by the presence of several somatic disorders that are prevalent in obese individuals such as diabetes type 2, hypertension, sleep apnea, dyslipidemia, or chronic pain.

Type 2 diabetes that is characterized by reduced insulin sensitivity and relative insulin deficiency is related to microvascular and macrovascular complications that may affect the brain (5–7). The underlying mechanisms of diabetes-related cognitive impairment, however, are difficult to establish due to the presence of several confounding somatic comorbidities. There exists evidence that cerebrovascular disease contributes substantially to decreased cognitive abilities in patients with diabetes type 2 (6, 7). Similarly, chronic elevation in blood pressure is common in obese persons and linked to an increased risk for alterations in the cerebral artery structure, vascular dementia, and Alzheimer-type neuropathy (8). Hypertension affects cerebral circulation that may adversely influence performance in cognitive tasks, in particular those assessing executive functions (9–11). Another vascular risk factor for cognitive dysfunction is dyslipidemia that is strongly associated with diabetes type 2 and hypertension in obese individuals (12, 13). Furthermore, sleep apnea has been associated with a higher risk for cognitive impairment (14, 15). Sleep fragmentation and cessation or reduction of breathing during sleep resulting in recurrent hypoxemia can contribute to structural and functional brain abnormalities that are related to cognitive dysfunction (16–18). In addition, there exists evidence for the negative influence of chronic pain on cognitive performance (19, 20). Possible mechanisms involved in pain-related cognitive impairment include the overlap in brain morphology, neurotransmitters, and other neural mediators that are involved in both pain processing and cognition, altered neuroplasticity, and dysregulated neurochemistry [for review see (20)].

There are other variables that can affect cognitive functioning. It is well known that morbidly obese individuals suffer from psychiatric disorders [e.g., (21–23)]. For example, depressive symptoms are prevalent in obese persons (24) and may account for their cognitive abilities (25). One of the most common psychiatric disorders reported in morbidly obese patients is binge eating disorder (BED) with prevalence rates up to 50% (23, 26–29). BED is characterized by experiencing loss of control while eating an unusual large amount of food and is therefore linked to food-related high impulsivity (30). Few studies have examined cognitive functions, in particular food-unrelated decision-making, in obese individuals with BED reporting mixed results. While one study found decision-making deficits in overweight and obese women with BED compared to those without (31) others did not (32, 33). The inconsistency between the studies could be caused by different cognitive tasks and by sampling characteristics [e.g., different body mass index (BMI) ranges].

Furthermore, higher education is often related to better performance in cognitive tasks (32, 34). Last but not least, there have been many reports regarding the inverse association between age and performance in cognitive tasks. The question at which age the cognitive decline starts, however, remains unresolved (34, 35).

The aim of the present study was to examine the relationship between neurocognitive performance and somatic disorders in obese patients taking into account BMI, depressive and eating disorder symptoms, and education. Based on the literature, we expected a reduced neurocognitive performance in obese individuals with any obesity-related somatic disorder compared to those without.

## Materials and Methods

### Participants and study design

Participation in the study was completely voluntarily. Inclusion criteria were a BMI ≥ 30 kg/m^2^, age between 18 and 65 years, and sufficient German language skills. Exclusion criteria were any neurological disorder, psychosis, dementia, current substance abuse, developmental or learning disorders, sensory impairments, and intellectual disability.

Between January 2011 and May 2012, 84 patients considering bariatric surgery who were seen for a routine preoperative psychiatric evaluation at the University Hospital Erlangen or the Hannover Medical School and 62 subjects from a psychosomatic inpatient unit in Bad Bramstedt were recruited. The total sample consisted of 146 patients (71.2% women, 28.8% men) with an average age of 39.8 years (SD = 11.4, Range 18–65). The majority (*N* = 113) suffered from obesity grade 3 (BMI ≥ 40 kg/m^2^), 26 individuals had obesity grade 2 (BMI: 35–39.9 kg/m^2^), and only 7 individuals had obesity grade 1 (BMI: 30–34.9 kg/m^2^). All participants gave written informed consent according to procedures approved by the Institutional Ethics Committees of the three study sites.

### Assessments

The participants provided information on education, height, and weight. Information on medication and the following somatic disorders was taken from patients’ charts of the surgical departments or the inpatient unit: hypertension, diabetes, sleep apnea, dyslipidemia, and pain disorder.

To measure eating disorder symptoms, the German version of the Eating Disorder Examination-Questionnaire (EDE-Q) (36) was used. This questionnaire consists of specific items to assess objective binge eating episodes (OBE; i.e., eating an objectively large amount of food with a sense of loss of control). Regular binge eating was defined as eight or more OBEs during the past 28 days.

The German version of the 9-item Patient Health Questionnaire depression scale (PHQ-9) (37) was administered in order to assess depressive symptoms. The PHQ-9 scores each of the nine DSM-IV criteria for depression from 0 (not at all) to 3 (nearly every day).

Neurocognitive functioning was measured by a computerized test battery that consisted of the following neurocognitive tasks.

The Corsi Block Tapping Test (CBT) was administered to assess visual attention and working memory (38). Participants had to reproduce several sequences of block tappings displayed only once by the computer. When the sequence was correctly copied, the number of cubes which had to be touched was increased step-wise. The outcome variable of this task was the total number of correct answers.

To assess verbal memory, we administered an auditory verbal learning task (wordlist) in which a list of 15 not associated words was presented five times. Participants were asked to reproduce as many words as possible after each of the five trials. The number of correctly recalled words per trial was registered and the total of all correct answers across the five trials was used as outcome variable.

The Trail Making Test-Part B (TMT-B) (39) was used to examine mental flexibility and the ability to switch attention. Participants had to tap numbers (1–9) and digits (A–I) in an alternating sequence as quickly and as accurately as possible within three trials. The mean time to completion per trial (in seconds) was used as dependent variable in this task.

The Stroop Test is a well known diagnostic tool for assessing selective attention and response inhibition (40). In this study, the answers were given not verbally but by pressing the corresponding button on the touch screen. The number of correctly identified words in the interference condition was used as dependent variable.

The Labyrinth Test was used to assess spatial memory, planning, and error utilization (41). Participants had to find a hidden path through a spatial maze. The task was completed after the hidden path was found two times without errors. The total number of errors during this task was used as dependent variable.

Finally, a four-disk version of the Tower of Hanoi (ToH) was used to measure planning abilities (42). The ratio between the number of ideal disk moves and the number of actually needed disk moves (effectivity) was the dependent variable.

### Data analysis

Analyses were performed using IBM SPSS Statistics v.20. The total sample was divided in a group without any somatic disorder and a group with at least one somatic disorder. Univariate analyses of variance (ANOVA) and χ^2^-tests were used to compare the groups in terms of sociodemographic variables, BMI and depression, as appropriate. In a second step, the groups were compared with regard to their performance in the neurocognitive tasks. These analyses were controlled for potential confounding variables that were found to be different between the groups in the aforementioned analyses (ANCOVA). The group comparisons were then repeated subsequently for specific somatic disorders (i.e., hypertension yes/no, diabetes yes/no, sleep apnea yes/no).

To investigate the relationship between the number of somatic disorders and the performance in neurocognitive tasks, we conducted Pearson correlations. Finally, to examine the association between neurocognitive performance and the presence of any somatic disorder we performed a set of logistic regression analyses adjusting for variables that differed between the two groups (age, gender, BMI).

The group comparisons regarding cognitive functioning and the regressions were made on the basis of Bonferroni-corrected significance levels.

## Results

### Somatic comorbidity, BMI, medication, sociodemographic characteristic, eating disorder symptoms, and depression

Based on patients’ charts, 67.1% of the total sample (*N* = 98) suffered from at least one somatic disorder (Soma^+^-group). Hypertension was present in 75 individuals (51.4%), type 2 diabetes in 34 participants (23.3%), 38 individuals had sleep apnea (26.0%), 16 suffered from dyslipidemia (11.0%), and 14 individuals reported having a chronic pain disorder (9.6%). The mean number of disorders in the Soma^+^-group ranged from 1 to 5 (*M* = 1.81, SD = 0.94). Almost half of that group reported suffering from only one somatic disorder (49%), 26.5% had two, and 20.4% three somatic disorders. The number of somatic disorders in the Soma^+^-group was positively related to age (*r* = 0.293, *p* < 0.01).

The groups did not differ in terms of any psychopharmacological treatment including neuroleptics, mood stabilizer, antidepressants, anxiolytics, stimulants, or anticonvulsive drugs [Soma^−^: 37.5% vs. Soma^+^: 45.4%, χ^2^(1) = 0.811, *p* = 0.368]. Many participants reported taking antidepressant medication [Soma^−^: 33.3% vs. Soma^+^: 43.3%, χ^2^(1) = 1.329, *p* = 0.249]. While only two participants in the Soma^−^-group took non-opiate pain medication, 81.4% of participants with somatic comorbidity had at least one of the following pharmacological treatments: insulin and/or non-insulin anti-diabetic medication, antihypertensive agents, opiate or non-opiate pain medication, or cholesterol lowering drugs.

As shown in Table 1, patients with any somatic disorder were substantially older and had a significantly higher BMI than those without somatic disorders. There were more male patients in the Soma^+^-group. The groups did not differ significantly from each other with regard to education.

**Table 1:** **Comparison of sociodemographic characteristics, BMI, eating disorder, and depressive symptoms between individuals without any somatic disorder (Soma^−^) and those with any somatic disorder (Soma^+^)**.

	Soma^−^ (*N* = 48)	Soma^+^ (*N* = 98)	Group comparison
		
	*M* (SD)	*M* (SD)	ANOVA
Age (years)	33.7 (9.8)	42.7 (11.0)	*F*(1, 144) = 23.008, *p* < 0.001
BMI	43.9 (8.4)	48.4 (8.3)	*F*(1, 144) = 9.483, *p* = 0.001
PHQ-9	10.6 (6.6)	9.4 (5.8)	*F*(1, 124) = 1.088, *p* = 0.299
EDE-Q	3.13 (1.05)	2.90 (1.00)	*F*(1, 130) = 1.538, *p* = 0.217

	***N* (%)**	***N* (%)**	**χ^2^-test**

**OBESITY GRADE**			
Grade I	4 (8.3)	3 (3.1)	χ^2^(2) = 14.904, *p* = 0.001
Grade II	16 (33.3)	10 (10.2)	
Grade III	28 (58.3)	85 (86.7)	
Gender, female	43 (89.6)	61 (62.2)	χ^2^(1) = 11.751, *p* = 0.001
**SCHOOL YEARS**			
≤9	9 (19.6)	33 (34.4)	χ^2^(3) = 4.029, *p* = 0.258
10	23 (50.0)	42 (43.8)	
11–13	9 (19.6)	11 (11.5)	
>13	5 (10.9)	10 (10.4)	

*PHQ-9, Patient Health Questionnaire, depression scale (data were available from 43 participants without any somatic disorder and 83 participants with at least one somatic disorder); EDE-Q, Eating Disorder Examination-Questionnaire (data were available from 44 participants without any somatic disorder and 88 participants with at least one somatic disorder)*.

According to the EDE-Q mean total scores which were available from 134 patients, we did not find any significant group differences in terms of eating disorder symptoms including binge eating. The mean number of OBEs during the past 28 days did not differ between the two groups [*M*_Soma+_ = 4.27, SD = 6.34 and *M*_Soma−_ = 4.78, SD = 7.18, *F*(1, 133) = 0.175, *p* = 0.676]. Regular binge eating was found in 21.3% of the Soma^+^ group and 28.9% of the Soma^−^ group [χ^2^(1) = 0.935, *p* = 0.334]. Information on depressive symptoms was available from 126 patients (86% of the total sample). The PHQ-9 data indicated a lack of significant group differences with respect to depression.

### Comparison of performance in neurocognitive tasks between patients without and with somatic disorders

As can be seen in Table 2, the results of the univariate ANOVA suggest differences between the Soma^−^ and the Soma^+^ groups in three of six tasks, particularly in the CBT, the wordlist, and the TMT-B. After controlling for gender, only the difference in the CBT remained significant. After adjusting for age all group differences disappeared completely (Table 2).

**Table 2:** **Comparison of performances in neurocognitive tasks between individuals without any somatic disorder (Soma^−^, *N* = 48) and those with any somatic disorder (Soma^+^, *N* = 98)**.

	Soma^−^	Soma^+^	Group comparison			
		
	*M* (SD)^a^	*M* (SD)^a^	ANOVA	ANCOVA controlled for age	ANCOVA controlled for gender	ANCOVA controlled for BMI
Corsi Block Tapping Test	7.31 (2.38)	5.64 (2.19)	*F*(1, 144) = 17.50^**^	*F*(1, 143) = 6.89	*F*(1, 142) = 10.39^*^	*F*(1, 143) = 16.34^**^
Wordlist	58.75 (8.23)	52.83 (8.92)	*F*(1, 143) = 14.85^**^	*F*(1, 142) = 4.69	*F*(1, 141) = 3.24	*F*(1, 142) = 13.35^**^
Trail Making Test, Part B	6792.06 (2216.35)	8420.36 (3321.52)	*F*(1, 143) = 9.44^*^	*F*(1, 142) = 0.95	*F*(1, 141) = 6.58	*F*(1, 142) = 11.49^**^
Stroop Test	19.85 (0.36)	19.58 (1.14)	*F*(1, 144) = 2.61	*F*(1, 143) = 0.03	*F*(1, 142) = 0.001	*F*(1, 143) = 4.59
Austin Maze	43.00 (35.90)	48.97 (30.50)	*F*(1, 136) = 1.04	*F*(1, 135) = 0.79	*F*(1, 134) = 0.08	*F*(1, 135) = 0.72
Tower of Hanoi	0.52 (0.22)	0.51 (0.23)	*F*(1, 143) = 0.02	*F*(1, 142) = 0.03	*F*(1, 141) = 0.22	*F*(1, 142) = 0.03

^a^unadjusted means and standard deviations; *p < 0.008 (0.05/6); **p < 0.001.

**Figure 1:**
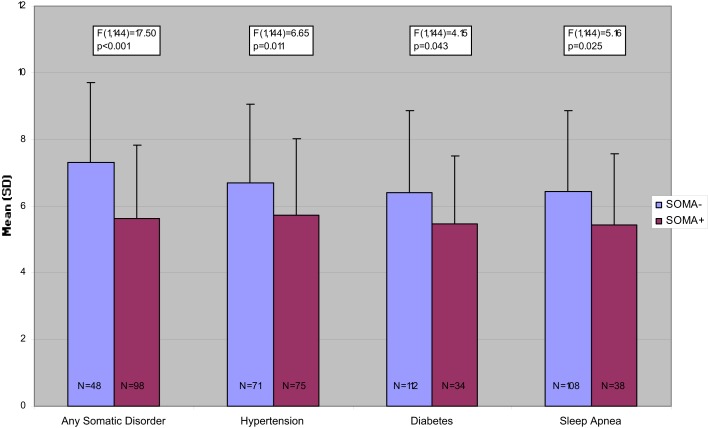
**Number of correct answers in the Corsi Block Tapping Task, unadjusted comparisons**.

**Figure 2:**
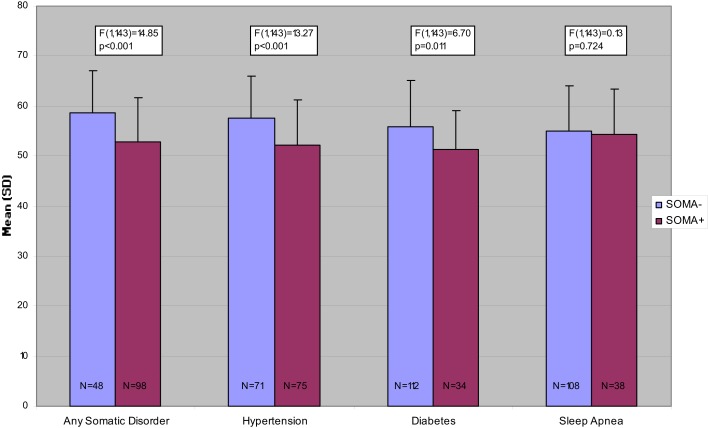
**Number of correctly recalled words in the Wordlist Task, unadjusted comparisons**.

**Figure 3:**
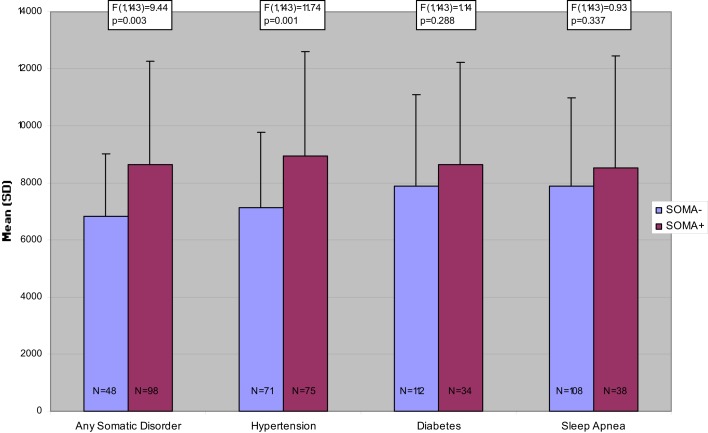
**Mean time to completion per trial in the Trail Making Test-Part B, unadjusted comparisons**.

Figures 1–3 present the unadjusted group differences of task performance in the CBT, the wordlist, and the TMT-B by the presence/absence of specific disorders, in particular hypertension, diabetes, and sleep apnea indicating the strongest effect on cognitive performance for hypertension. Similarly to the aforementioned findings, after controlling for age the differences were no longer significant (data not reported here but available upon request).

Due to the relatively low prevalence of dyslipidemia and pain disorder in the present sample we did not conduct group comparisons based on the absence or presence of these conditions.

### Relationship between the number of comorbid disorders and performance in neurocognitive tasks

Pearson correlations between the number of somatic disorders and neurocognitive performance yielded significant negative correlations with regard to the CBT (*r* = −0.273, *p* < 0.01), the wordlist (*r* = −0.309, *p* < 0.001), and the TMT-B (*r* = −0.315, *p* < 0.001) but not the other tasks. Since the number of somatic disorders was positively correlated with age (*r* = 0.452, *p* < 0.001), we conducted additional partial correlations controlling for age. After this, the correlations mentioned above were no longer significant (CBT: *r* = −0.111, ns; wordlist: *r* = −0.126, ns; TMT-B: *r* = 0.111, ns).

### Association between performance in neurocognitive tasks and somatic comorbidity

Table 3 summarizes the results of hierarchical regression analyses with the performance in the specific neurocognitive tasks as dependent variables. Age, gender, and BMI were entered into block 1 of the regressions as control variables. The variable “presence of any somatic disorder” was entered in a second step. Changes in predictive ability (*R*^2^) were examined to determine significance. As shown in Table 3, only lower age was associated with more correct answers in the CBT and with a higher number of correctly recalled words in the wordlist task. Furthermore, age was positively related to an increased time to completion in the TMT-B.

**Table 3:** **Summary of linear regression models concerning the association between performance in neurocognitive tasks and the presence of any comorbid somatic disorder, controlled for age, gender, and BMI**.

	Corsi Block Tapping Test		Wordlist		Trail Making Test, part B	
	β	*R*^2^	β	*R*^2^	β	*R*^2^
**Step 1**
Age	−0.39^**^	0.17^**^	−0.40^**^	0.22^**^	0.48^**^	0.25^**^
Gender	0.05		0.19^*^		−0.10	
BMI	−0.07		−0.08		−0.08	
**Step 2**
Age	−0.32^**^	0.20^**^	−0.36^**^	0.23^**^	0.45^**^	0.26^**^
Gender	−0.00		0.16		−0.08	
BMI	−0.03		−0.05		−0.10	
Somatic comorbidity	−0.23		−0.11		0.08	

*p < 0.017 (0.05/3); **p < 0.001.

## Discussion

In the present sample, individuals with any somatic disorder were about 10 years older and reported significantly higher BMIs than those without any somatic disorder. Moreover, age was positively related to the number of obesity-related somatic disorders in the Soma^+^-group.

At the first glance, our results concerning cognitive performance suggested working and verbal memory deficits (CBT, wordlist) and poorer mental flexibility (TMT-B) in obese individuals with obesity-related somatic comorbidity. While controlling for BMI did not impact the results substantially, the group differences disappeared after adjusting for age and gender. Given the results of the regression analyses with the performance in the specific neurocognitive tasks as dependent variables and age, gender, BMI, and somatic comorbidity as independent variables, the better task performance in the Soma^−^-group seems mainly driven by younger age. The groups did not differ in terms of education, depressive symptoms, or eating pathology. Hence, we may assume that the findings were not confounded by these variables. Taken together, it appears that worse performance in three of the six cognitive tasks were rather a problem of older age than of somatic comorbidity. This is in line with previous research demonstrating age-related cognitive decline (34, 35).

The findings do not support the assumption that somatic comorbidity is related to a higher risk for cognitive impairment in obese individuals. This is surprising given earlier reports on the association between cognitive dysfunction and somatic comorbidity. Though, many of those did not focus on obese individuals [e.g., (10, 15, 19)]. The discrepancy between our and previous findings reporting cognitive decline in overweight or obese individuals with somatic comorbidity might be explained by differences in test selection. Moreover, differences in sampling may account for the discrepant findings. For example, other studies had included either substantially younger [e.g., (17)] or older [e.g., (9)] individuals or those with lower BMIs [e.g., (7)]. It is certainly possible that the lack of group differences in the present investigation was related to a ceiling effect in both the Soma^+^ and the Soma^−^ group given the high BMI range. According to previous reports, obese individuals suffer from impaired cognitive function compared to normal-weight controls [e.g., (3, 4, 43, 44)]. However, the present investigation did not include a normal-weight control group that is a shortcoming.

On the other hand, our results are consistent with other reports of lacking differences in cognitive function between individuals with and without somatic comorbidity. Recently, Singh-Manou et al. (45) examined the association between midlife obesity, metabolic abnormalities (i.e., dyslipidemia, hypertension, hyperglycemia), and cognitive decline in early old age in a large longitudinal study (*N* = 6401). The sample consisted of 582 obese adults. In this study, the metabolically healthy obese participants did not have a better cognitive profile than individuals with metabolic abnormalities. Also, no significant differences were found between the two groups with regard to cognitive decline over a 10 years period.

There are some other limitations that need to be acknowledged. First, the diagnosis of both depressive symptoms and binge eating based on questionnaires instead of a standardized interview is concerning. Second, medication for somatic disorders could have influenced the task performance. Also, the group with somatic comorbidity is twice as large as the group without any somatic disorder that might have biased the results.

In conclusion, our findings indicate that in some obese patients increasing age may not only be accompanied by an increase of obesity severity and by more obesity-related somatic disorders but also by poorer cognitive functioning. Further studies should include control groups matched for age, gender, and education to examine whether somatic comorbidity may add a negative impact on cognition above the effect of age.

## Conflict of Interest Statement

The authors declare that the research was conducted in the absence of any commercial or financial relationships that could be construed as a potential conflict of interest.
